# Preoperative dual antiplatelet therapy increases risk after urgent coronary bypass surgery: A Netherlands heart registration study

**DOI:** 10.1016/j.xjon.2025.09.045

**Published:** 2025-10-13

**Authors:** Heleen J.C.L. Apostel, Maaike M. Roefs, Amon Heijne, Ewald M. Bronkhorst, Edgar J. Daeter, Wilson W.L. Li, Cardiothoracic Surgery and PCI Registration Committee of the Netherlands Heart Registration

**Affiliations:** a Department of Anesthesia, Pain and Palliative Medicine, Radboud University Nijmegen Medical Center, Nijmegen, the Netherlands; b Netherlands Heart Registration, Utrecht, the Netherlands; c Department of Cardiothoracic Anesthesiology, Radboud University Nijmegen Medical Center, Nijmegen, the Netherlands; d Department of IQ Health, Radboud University, Nijmegen, the Netherlands; e Department of Cardiothoracic Surgery, St Antonius Hospital, Nieuwegein, the Netherlands; f Department of Cardiothoracic Surgery, Radboud University Nijmegen Medical Center, Nijmegen, the Netherlands

**Keywords:** coronary artery bypass grafting, dual antiplatelet therapy, acute coronary syndrome, myocardial infarction, bleeding, surgical outcomes, nationwide registry

## Abstract

**Objective:**

Dual antiplatelet therapy (DAPT) is standard care after acute coronary syndrome, but its perioperative management before urgent coronary artery bypass grafting (CABG) remains controversial. By using data from the Netherlands Heart Registration, a nationwide Dutch registry, we sought to assess the impact of recent preoperative DAPT on surgical and postoperative outcomes in patients undergoing urgent CABG after acute coronary syndrome.

**Methods:**

In this multicenter retrospective cohort study, 6913 patients undergoing urgent isolated CABG within 90 days of acute coronary syndrome were analyzed. Patients receiving DAPT (aspirin + P2Y12 inhibitor within 48 hours preoperatively) were compared with those on aspirin alone. Propensity score matching and multivariable logistic regression were used to adjust for confounding.

**Results:**

Recent DAPT use was independently associated with increased perioperative bleeding complications, including greater rates of reintervention (odds ratio [OR], 1.78), transfusion (OR, 1.85), and surgical mortality (OR, 2.02). Considerable interhospital variation in DAPT use (12%-84%) underscores inconsistent practices across Dutch cardiac surgery centers.

**Conclusions:**

Recent DAPT before urgent CABG is independently associated with significantly increased perioperative bleeding risk, transfusion requirements, and mortality. The substantial interhospital variation in DAPT use across Dutch cardiac surgery centers further underscores the need for standardized, evidence-based guidelines to optimize antiplatelet management in high-risk patients with coronary syndrome requiring surgical revascularization.

## Graphical Abstract

**Figure:**
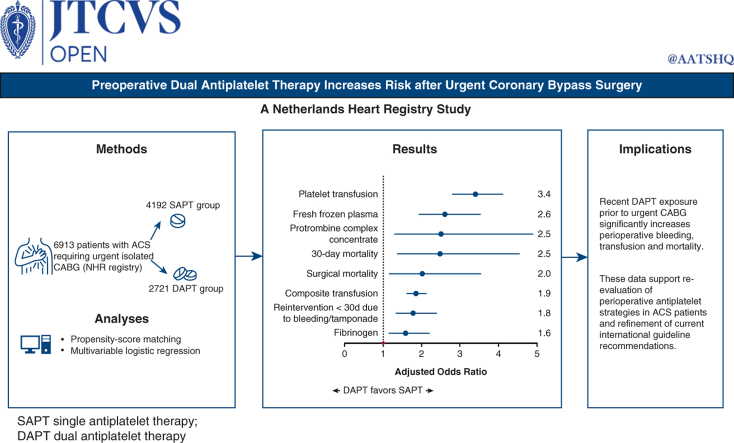

**Figure:**
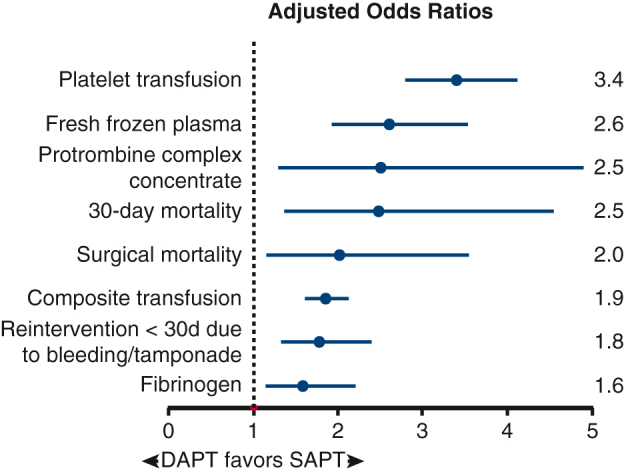
Risk of bleeding and adverse outcomes with DAPT before urgent CABG surgery.

**Central Message**

Recent DAPT exposure before urgent CABG increases bleeding, transfusion needs, and short-term mortality, supporting revision of antiplatelet strategies and guidelines in ACS patients.

**Perspective**

In this large nationwide cohort, recent DAPT exposure before urgent CABG independently increased bleeding-related reinterventions, transfusion, and mortality. These findings challenge the adequacy of current guideline recommendations and advocate for more nuanced, evidence-based perioperative antiplatelet protocols tailored to urgency, risk profile, and antiplatelet agent used.

Dual antiplatelet therapy (DAPT), which combines aspirin with a P2Y12 inhibitor (eg, clopidogrel or ticagrelor), is recommended in all patients after acute coronary syndrome (ACS) for a period of at least 12 months by the current American Heart Association/American College of Cardiology and European Society of Cardiology guidelines.^1^^,^^2^ A significant proportion of these patients after recent ACS, that is, 4.5% to 10%,^3^^,^^4^ will have to undergo urgent coronary artery bypass grafting (CABG). For these patients, the optimal perioperative antithrombotic regimen remains debated. Although DAPT has shown undeniable benefits in preventing further major adverse cardiovascular events in patients with ACS,^5^ concerns have emerged regarding the potential impact of preoperative DAPT on the outcomes of CABG, especially in terms of perioperative bleeding and postoperative complications.^6^^,^^7^ In this context, perioperative bleeding not only increases the need for blood transfusion but also increases the risk of reintervention. Re-exploration for bleeding is associated with significantly increased morbidity and a marked increase in mortality.^8^^,^^9^

International guidelines provide differing recommendations regarding the management of DAPT in the context of cardiac surgery, with substantial variation especially for urgent cases. In line with these recommendations, real-world practice remains heterogenous, and evidence guiding DAPT management in urgent CABG is limited. Many studies focus primarily on elective procedures or in limited cohorts,^10^^,^^11^ leaving an important knowledge gap in patients with high-risk ACS undergoing urgent surgical revascularization. Moreover, intercenter variation in DAPT use suggests a lack of consensus and underlines the need for further clinical data to uniform practice.^12^^,^^13^

In this multicenter nationwide analysis from the Netherlands, we aimed to evaluate the impact of recent preoperative DAPT on bleeding-related outcomes in patients undergoing urgent isolated CABG. We focused on reinterventions, transfusion requirements, and postoperative complications including mortality, hypothesizing that recent DAPT exposure is independently associated with adverse perioperative outcomes.

## Methods

### Study Design and Data

This retrospective multicenter cohort study used prospectively collected data from the Netherlands Heart Registration (NHR). This is a mandatory nationwide physician-driven and patient-focused quality registry of patients undergoing cardiac surgery, designed to improve the outcomes of patients with cardiac disease who undergo surgery.^14^^,^^15^ All cardiac surgery centers in the Netherlands participate in this registry. Participating centers enter data in a secure online system, with regular quality checks.^16^ Data were obtained and analyzed with approval from the NHR registry board. The study was approved by the institutional review board MEC-U (W19.270; January 2, 2020) and conducted in agreement with the principles of the Declaration of Helsinki. A waiver for informed consent for analysis with the data of the NHR data registry was obtained.

### Study Population

We identified all adult patients who underwent urgent, isolated CABG from January 2017 until December 2022. Isolated CABG was defined as a primary procedure without concomitant cardiac interventions. Urgent surgery was defined as nonelective and performed during the same hospitalization, without the possibility of safe discharge before surgery. Only patients with a recent ACS within 90 days before surgery were included. Patients who underwent emergency surgery were excluded.

**Figure 1:**
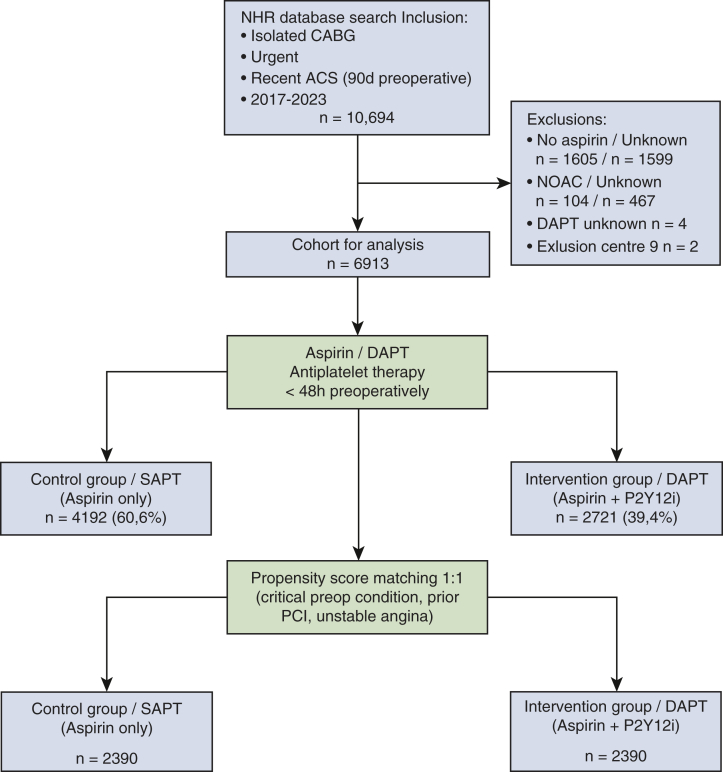
Patient selection flowchart and treatment stratification. *DAPT*, Dual antiplatelet therapy; *SAPT*, single antiplatelet therapy.

Patients were excluded if they had missing data on preoperative antiplatelet therapy or were treated with non-vitamin K oral anticoagulants. In addition, center 9 was excluded from the analysis because of the submission of only 2 patients, which precludes meaningful analysis and could distort statistical modeling and intercenter comparisons (Figure 1).

### Grouping and Antiplatelet Therapy

Patients were classified into 2 groups on the basis of antiplatelet therapy within 48 hours before surgery: single antiplatelet therapy (SAPT) with aspirin only and DAPT with additional clopidogrel, prasugrel, or ticagrelor.

### Patients and Outcome Characteristics

All patients underwent urgent, isolated, primary CABG, either on- or off-pump according to local clinical practices. Baseline patient characteristics included the raw European System for Cardiac Operative Risk Evaluation (EuroSCORE) II items. In addition, the use of CPB, CPB time, center distribution, and outcome variables such as reinterventions, blood transfusion, and postoperative morbidity and mortality data were recorded.

Multivessel disease was defined as ≥70% stenosis in 2 or more major coronary arteries (right coronary artery, left anterior descending artery, left circumflex artery) or their first-order branches; left main stenosis was classified as 2-vessel disease, and single-vessel disease as ≥70% stenosis in only 1 vessel.

We defined 2 primary outcomes: first, the number of reinterventions within 30 days postoperatively caused by bleeding or tamponade; second, a composite transfusion-related end point including any transfusion of red blood cells, platelets, fresh-frozen plasma, and/or administration of coagulation products (eg, fibrinogen, prothrombin complex concentrate).

The secondary outcomes consisted of the individual rates of administered blood and coagulation products; in-hospital mortality, 30-day mortality, together defined as surgical mortality, 1-year mortality; postoperative complications including cerebrovascular events, infections, respiratory or renal failure, prolonged mechanical ventilation exceeding 24 hours, and readmission to the intensive care unit.

Reintervention within 30 days was defined as any unplanned surgical or interventional procedure after sternal closure as the result of complications of the index operation. The registry records reinterventions within 30 days after surgery, without details on exact timing. Reinterventions are coded by underlying cause, including bleeding/tamponade, cardiac complications, or other indications.

### Statistical Analysis

Descriptive statistics were used to summarize patient characteristics and outcomes. Categorical variables are presented as percentages (%) and continuous variables are reported as mean (standard deviation) or median [interquartile range], on the basis of distribution. Given the observational nature of the study, no statistical comparisons or *P* values were reported for baseline variables, in accordance with current reporting recommendations.

Variables that were considered clinically relevant and differed meaningfully between the SAPT and the DAPT groups were included in a propensity score matching (PSM) analysis to reduce confounding. To reduce potential confounding, 1:1 PSM was performed applying matching on clinically relevant imbalanced variables: unstable angina, previous percutaneous coronary intervention (PCI), critical preoperative condition, and use of CPB. Because all these variables were dichotomous, exact matching could be performed. We explored adding recent PCI (<3 months), but this led to loss of a substantial number of patients and did not alter the results; therefore, this variable was not retained in the PSM. Matching was done using the MatchIt library.

Multivariable logistic regression was used to assess the association between DAPT and the main perioperative outcomes: reintervention as the result of bleeding and/or tamponade, composite transfusion, 30-day mortality, surgical mortality, and 1-year mortality. A random intercept for each participating center was included to account for clustering, estimating population-level effects while adjusting for center-specific variation. To reduce variability and thus increases precision of the estimate, EuroSCORE II and recent PCI (<3 months) were added as covariates to the model. Results are reported as odds ratios (OR) with 95% confidence intervals (CIs) and 2-sided *P* values. All analyses were performed using R (version 4.3.1; R Foundation for Statistical Computing) and IBM SPSS statistics (version 28.0, IBM Corp).

## Results

**Figure 2:**
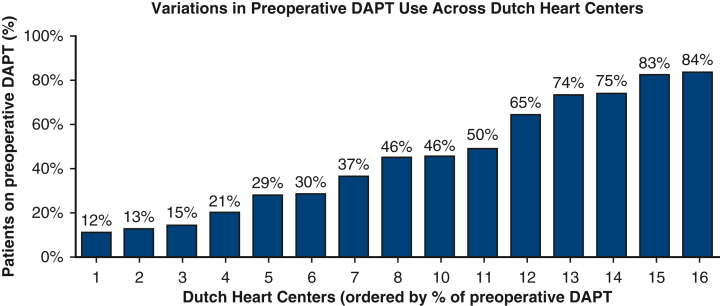
Adjusted odds ratios (ORs) for perioperative outcomes in patients undergoing urgent CABG after recent ACS (DAPT vs SAPT). Forest plot showing adjusted ORs with 95% confidence intervals for key perioperative outcomes comparing DAPT with SAPT in patients undergoing urgent CABG after ACS. All confidence intervals not containing the value 1, indicated by the *dashed vertical line*, show a statistical significant effect (*P* < .05). Outcomes include reintervention, transfusion requirement (composite and individual blood products), infection rates, ICU readmission, respiratory insufficiency, CVA, and short- and long-term mortality. Estimates are derived from a propensity score–matched cohort and further adjusted using multivariable logistic regression for EuroSCORE II and treatment center to account for baseline surgical risk and institutional practice variation. For reoperation during hospitalization, low event counts precluded full adjustment. *CABG*, Coronary artery bypass grafting; *ACS*, acute coronary syndrome; *DAPT*, dual antiplatelet therapy; *SAPT*, single antiplatelet therapy; *ICU*, intensive care unit; *CVA*, cerebrovascular accident; *EuroSCORE*, European System for Cardiac Operative Risk Evaluation score.

In total, 10,694 patients who met the inclusion criteria and were operated on in 1 of the 16 Dutch heart centers were identified in the NHR database. After exclusion of patients with no or unknown aspirin use (n = 3204), unknown DAPT use (n = 4), those treated with non-vitamin K oral anticoagulants or with unknown anticoagulant status (n = 571), and those from center 9 (n = 2), 6913 patients remained for analysis (Figure 2). Aspirin use is a mandatory variable in the Netherlands Heart Registration. Of the excluded patients, 1605 were explicitly recorded as not receiving aspirin, and 1599 had missing (“unknown”) aspirin status. Missing values are likely related to incomplete or delayed data entry at the institutional level.

On the basis of preoperative antiplatelet therapy within 48 hours before surgery, 4192 patients were (60.5%) were classified in the SAPT group and 2721 patients (39.5%) in the DAPT group. As the result of missing data, the total number per examined parameter will vary. Therefore, denominators are given per variable in the tables when appropriate.

### Center Variation

**Figure 3:**
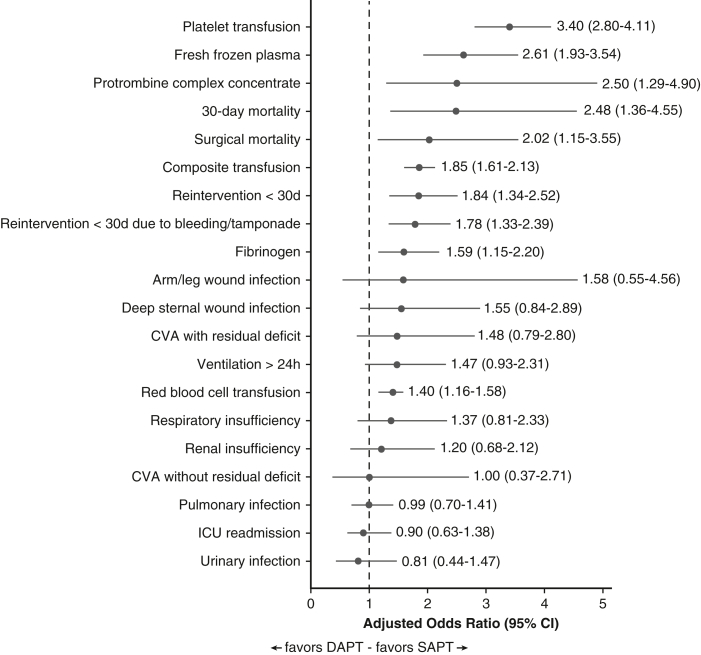
Variation in preoperative DAPT use across Dutch heart centers. Each *bar* represents one heart center, ordered by increasing percentage of preoperative DAPT use. *DAPT*, Dual antiplatelet therapy; *SAPT*, single antiplatelet therapy; *ACS*, acute coronary syndrome; *CABG*, coronary artery bypass grafting; *NHR*, Netherlands Heart Registration.

A significant variation in the preoperative use of DAPT was observed across Dutch heart centers as shown by the considerable spread in DAPT use preoperatively, ranging from 12.3% to 84.4%, *P* < .001 (Figure 3).

### Baseline Characteristics

The patients in both groups were comparable on most baseline clinical parameters, with a greater proportion of unstable angina, CCS class IV, preoperative PCIs, and critical preoperative condition in DAPT (Table 1). The majority of procedures were performed with the use of CPB. Mean CPB time was not clinically significantly longer in the DAPT group compared with the SAPT group (73 minutes vs 79 minutes). Off-pump CABG was performed in 15.3% of patients overall and was similar in both groups (DAPT vs SAPT: 14.2% vs 16.1%, Table 1).

**Table 1:** Baseline patient and procedural characteristics in the SAPT and DAPT groups

Characteristic	SAPT, all data	DAPT, all data	SAPT after PSM	DAPT after PSM
Age, y, mean (SD)	67 (9)	66 (10)	67 (9)	67 (10)
Female gender, n (%)	803/4192 (19.2)	480/2721 (17.6)	459/2390 (19.2)	421/2390 (17.6)
BMI, kg/m^2^, mean (SD)	27.5 (4.5)	27.4 (4.1)	27.4 (4)	27.4 (4.1)
Diabetes mellitus, n (%)	1108/4184 (26.5)	716/2721 (26.3)	669/2384 (28.1)	633/2387 (26.5)
Creatinine, μmol/L, median [IQR] and mean (SD)	85 [74-98]	84 [72-98]	91 (41)	91 (43)
eGFR, mL/min/1.73 m^2^, mean (SD)	75 (20)	77 (21)	75 (20)	77 (21)
Dialysis, n (%)	16/4191 (0.4)	10/2720 (0.4)	13/2390 (0.5)	8/2390 (0.3)
Preoperative hemoglobin mmol/L, mean (SD)	8.6 (1.1)	8.5 (1.1)	8.5 (1.1)	8.5 (1.2)
LVEF, %, median [IQR] and mean (SD)	55 [40-55]	55 [40-55]	50 (9)	50 (10)
PAP systolic, mm Hg, mean (SD)	25 (4)	26 (4)	25 (4)	26 (4)
Unstable angina, n (%)	329/4192 (7.8)	265/2713 (9.8)	247/2390 (10.3)	247/2390 (10.3)
Multivessel disease, n (%)	3471/3695 (93.3)	2228/2398 (92.9)	1982/2101 (94.3)	1988/2113 (94.1)
EuroSCORE I, %, median [IQR] and mean (SD)	3.95 [2.30-6.54]	3.95 [2.28-6.71]	5.38 (4.7)	5.48 (5.1)
EuroSCORE II, %, median [IQR] and mean (SD)	1.87 [1.28-2.81]	1.79 [1.21-2.71]	2.50 (2.14)	2.47 (2.34)
Atrial fibrillation, n (%)	221/3658 (6.0)	128/2491 (5.1)	151/2100 (6.5)	111/2170 (5.1)
CCS class IV, n (%)	431/4081 (10.6)	372/2694 (13.8)	283/2330 (12.1)	334/2382 (11.8)
Critical preoperative condition, n (%)	67/4192 (1.6)	88/2721 (3.2)	60/2390 (2.5)	60/2390 (2.5)
Previous CVA, n (%)	157/4086 (3.8)	100/2582 (3.9)	93/2335 (4)	86/2260 (3.6)
Chronic lung disease, n (%)	338/4192 (8.1)	213/2721 (7.8)	193/2390 (8.1)	185/2390 (7.7)
Extracardiac arteriopathy, n (%)	395/4191 (9.4)	268/2718 (9.9)	232/2389 (9.7)	242/2387 (10.1)
Neurologic disease, n (%)	71/4086 (1.7)	51/2713 (1.9)	40/2320 (1.7)	44/2383 (1.8)
Previous PCI, n (%)	845/4096 (20.6)	910/2485 (36.6)	833/2390 (34.9)	833/2390 (34.9)
PCI <3 mo before CABG, n (%)	260/4192 (6.2)	644/2721 (23.7)	254/2390 (10.6)	547/2390 (22.9)
Off-pump CABG, n (%)	675/4183 (16.1)	385/2709 (14.2)	343/2390 (14.4)	343/2390 (14.4)
CPB time, min, median [IQR] and mean (SD)	75 [53-100]	80 [57-106]	73 (44)	79 (45)

Demographics, comorbidities, preoperative risk scores, and procedural details are shown for patients undergoing urgent isolated CABG. Values are presented as n (%) or mean (SD), median [IQR], as appropriate. For “female gender”, “diabetes mellitus” and “dialysis”, the two numbers indicate the number of patients with the given characteristic and the total number of patients in that group, respectively, with the corresponding percentage shown in parentheses (n/total, %). *SAPT*, Single antiplatelet therapy; *DAPT*, dual antiplatelet therapy; *PSM*, propensity score matching; *SD*, standard deviation; *BMI*, body mass index; *IQR*, interquartile range; *eGFR*, estimated glomerular filtration rate; *LVEF*, Left ventricle ejection fraction; *PAP*, Pulmonary artery pressure; *EuroSCORE*, European System for Cardiac Operative Risk Evaluation score; *CCS*, Canadian Cardiovascular Society; *CVA*, cerebrovascular accident; *PCI*, percutaneous coronary intervention; *CABG*, coronary artery bypass graft.

### Clinical Outcomes After PSM

After PSM, 2390 patients were included in both the SAPT and DAPT groups. Matching was performed on unstable angina, previous PCI, critical preoperative condition, and use of CPB. The primary outcome, “reintervention within 30 days due to bleeding/tamponade,” was significantly more common in the DAPT group compared with SAPT group (7.1% vs 3.7%, Table 2). The majority of these reinterventions were bleeding or tamponade-related (DAPT group vs SAPT group 7.4% vs 3.1%; Table 2).

**Table 2:** Postoperative reinterventions and transfusion-related outcomes after PSM in patients undergoing urgent isolated CABG

Outcome	SAPT after PSM	DAPT after PSM	Difference DAPT − SAPT (95% CI)	*P* value
Reintervention	87/2356 (3.7%)	165/2330 (7.1%)	3.2% (2.0-4.8)	<.001
Reintervention due to bleeding/tamponade	73/2356 (3.1%)	149/2330 (6.3%)	3.2% (1.8-4.6)	<.001
Composite transfusion	639/2371 (27.0%)	1057/2353 (44.9%)	17.9% (15.3-20.5)	<.001
Red blood cell transfusion	495/2390 (20.7%)	712/2389 (29.8%)	9.1% (6.7-11.5)	<.001
Platelet transfusion	178/2390 (7.4%)	581/2389 (24.3%)	16.9% (14.8-19)	<.001
Fibrinogen administration	91/2362 (3.9%)	138/2306 (6.0%)	2.1% (0.8-3.4)	<.001
Prothrombin complex concentrate	11/2362 (0.5%)	35/2306 (1.5%)	1.0% (0.4-1.6)	<.001

*SAPT*, Single antiplatelet therapy; *PSM*, propensity score matching; *DAPT*, dual antiplatelet therapy; *CABG*, coronary artery bypass grafting.

The composite transfusion end point occurred in 44.9% of patients in the DAPT group, compared with 27.0% in the SAPT group (Table 2). Among individual transfusion components, red blood cell transfusion was required in 29.8% of patients who received DAPT versus 20.7% in the SAPT group. Platelet transfusion occurred in 24.3% of patients who received DAPT compared with 7.4% of those who received SAPT. Fibrinogen administration was more common in the DAPT group (6.0% vs 3.9%), as was the use of prothrombin complex concentrate (1.5% vs 0.5%) and fresh-frozen plasma (7.7% vs 2.6%).

Secondary outcome measures concerning mortality demonstrated a greater 30-day mortality in the DAPT group (1.6% vs 0.8%; Table 3) and a greater surgical mortality (1.7% vs 0.9%; Table 3). This suggests an increased short-term mortality associated with DAPT. There were no statistically significant differences in secondary outcomes between groups regarding deep sternal wound infection, pulmonary and urinary infections, limb wound infections, intensive care unit readmission, or renal insufficiency (Table 3).

**Table 3:** Postoperative outcomes by treatment group before and after propensity score matching

Outcome	SAPT after PSM	DAPT after PSM	Difference DAPT-SAPT (95% CI)	*P* value
In-hospital mortality	17/2390 (0.7%)	29/2390 (1.2%)	0.5% (0.1-1.1)	.075
30-d mortality	18/2371 (0.8%)	38/2339 (1.6%)	0.8% (0.2-1.5)	.006
Surgical mortality	20/2368 (0.8%)	38/2338 (1.6%)	0.8% (0.1-1.4)	.015
Deep sternal wound infection	22/2351 (0.9%)	27/2329 (1.2%)	0.3% (−0.4 to 0.8)	.453
Pulmonary infection	71/2382 (3%)	70/2351 (3%)	0.0% (−1.0 to 1.0)	.995
Urinary infection	28/2382 (1.2%)	24/2351 (1.0%)	−0.2% (−0.7 to 0.4)	.610
Respiratory insufficiency	25/2381 (1.0%)	43/2360 (1.8%)	0.8% (0.1-1.4)	.025
Ventilation >24 h	39/2382 (1.6%)	60/2381 (2.5%)	0.9% (0.7-1.7)	.032
Readmission ICU	56/2226 (2.5%)	67/2294 (2.9%)	0.4% (−0.5 to 1.4)	.403
CVA without residual deficit	9/2385 (0.4%)	8/2381 (0.3%)	0.0% (−0.4 to 0.3)	.811
CVA with residual defect	19/2385 (0.8%)	23/2381 (1%)	0.2% (−0.4 to 0.7)	.532
Renal insufficiency	17/2390 (0.7%)	29/2390 (1.2%)	0.5 (−0.3 to 1.2)	.588

Data are presented after 1:1 PSM. Outcomes are grouped into 4 domains: mortality, infectious complications, respiratory and ventilatory support, and neurologic, renal, and ICU-related events. PSM was used to balance baseline characteristics, and outcome frequencies are reported as count/total (%). *SAPT*, Single antiplatelet therapy; *PSM*, propensity score matching; *DAPT*, dual antiplatelet therapy; *CI*, confidence interval; *ICU*, intensive care unit, *CVA*, cerebrovascular accident.

### Multivariable Logistic Regression Analysis

In the propensity score−matched cohort, multivariable logistic regression adjusted for EuroSCORE II, recent PCI (<3 months), and treatment center (as random effect) demonstrated that preoperative use of DAPT was independently associated with several adverse perioperative outcomes (Figure 2).

First, DAPT remained independently associated with a greater likelihood of reintervention within 30 days as the result of bleeding/tamponade (OR, 1.78; 95% CI, 1.33-2.39, *P* < .001) and composite transfusion end point (OR, 1.85; 95% CI, 1.61-2.13, *P* < .001). All individual transfusion components, including red blood cells, platelets, fresh-frozen plasma, and coagulation products such as fibrinogen and prothrombin complex concentrate, were also more frequently administered in the DAPT group.

Second, DAPT use was significantly associated with greater 30-day mortality (OR, 2.48; 95% CI, 1.36-4.55, *P* = .003) and surgical mortality (OR, 2.02; 95% CI, 1.15-3.55, *P* = .01). No significant associations were found between DAPT and infectious complications and intensive care unit readmission.

## Discussion

In this large, Dutch nationwide cohort study of patients undergoing urgent isolated CABG after recent ACS, preoperative exposure to DAPT within 48 hours was independently associated with substantially increased perioperative risk. Multivariable logistic regression demonstrated that DAPT was independently associated with significantly increased risk of adverse outcomes, including a 78% greater risk of bleeding-related reintervention, nearly doubled transfusion requirements, and more than a 2-fold increase in surgical mortality. These findings likely reflect the hemostatic challenges of operating under DAPT, which can amplify perioperative bleeding risk, lead to additional surgical interventions, and contribute to transfusion needs, postoperative morbidity, and mortality.

Our results align with previous studies reporting elevated bleeding risk and perioperative complications in patients receiving DAPT before surgery^10^^,^^11^^,^^17^ and complement a recent meta-analysis by Schoerghuber and colleagues,^18^ which showed that guideline-conforming discontinuation of P2Y12 inhibitors (≥5 days for clopidogrel, ≥3 days for ticagrelor) reduced severe bleeding by 50% without increasing the risk of 30-day mortality or postoperative ischemic events. However, the analysis by Schoerghuber and colleagues included a broad range of clinical contexts and institutional practices. In contrast, our study uniquely reflects a uniform, contemporary national cohort of patients undergoing urgent CABG, providing real-world insights into the perioperative consequences of recent DAPT exposure.

Although our findings highlight the bleeding-related risks of preoperative DAPT exposure, concerns have also been raised regarding the potential for suboptimal antiplatelet therapy during the preoperative waiting period to precipitate recurrent ischemic events in patients with recent ACS. Yet, data suggest that the actual incidence of such perioperative thrombotic complications during temporary DAPT discontinuation is low, particularly when surgery is performed within a controlled, guideline-conforming time frame.^16^^,^^17^ Unfortunately, ischemic events during the wait for surgery or a rescue PCI are not registered within the NHR registry.

In addition, we observed substantial intercenter variability in preoperative DAPT use across Dutch heart centers (ranging from 12% to 84%), despite comparable patient profiles and surgical indications. This mirrors findings from earlier studies, such as those by Hensley and colleagues^12^ and Janssen and colleagues^13^ and reflects a lack of consensus in perioperative antiplatelet management. The observed variation appears more driven by institutional preferences and local protocols than by evidence-based practice, emphasizing the need for standardized, guideline-supported strategies.

Current European Society of Cardiology and American Heart Association/American College of Cardiology guidelines recommend early initiation of DAPT in ACS,^1^^,^^2^^,^^11^ yet remain vague on perioperative management when urgent CABG is needed. The current class I recommendation to withhold P2Y12 inhibitors for at least 24 hours in urgent CABG may be insufficient. Our findings—and those from recent meta-analyses—suggest greater safety with longer drug discontinuation periods. Future guidelines should consider stratifying recommendations based on bleeding and thrombotic risk.

Beyond clinical outcomes, the increased need for reinterventions and blood products in patients treated with DAPT may also translate into substantial economic burden. Previous studies have shown that bleeding complications and transfusion are among the primary drivers of cost in cardiac surgery.^19^ The greater incidence of these events suggests that preoperative DAPT exposure not only impacts clinical safety but may also significantly increase health care resource use and overall cost of care.

In summary, this study demonstrates that DAPT exposure within 48 hours of urgent CABG is associated with significantly increased risks of bleeding, transfusion, reintervention, and mortality. These findings, in conjunction with recent meta-analytic data, support a more individualized and cautious approach to preoperative antiplatelet management in this high-risk population.

### Limitations

This study has several limitations that should be addressed. First, its retrospective observational design inherently limits the ability to establish causal relationships. Despite the use of PSM and multivariable adjustment, residual confounding from unmeasured variables cannot be excluded.

Second, although the study included a large national cohort, detailed information on the exact timing of P2Y12 inhibitor discontinuation was not available. Grouping was based on antiplatelet use within 48 hours before surgery, which may not fully capture pharmacodynamic variability or actual platelet inhibition at the time of surgery. Furthermore, data on platelet function testing were not available.

Third, the registry records reinterventions and transfusions within 30 days after surgery, without details on exact timing. As a result, we were unable to distinguish between early (<72 hours) and late (>72 hours) events. Finally, although the study adjusted for key clinical variables and center effects, granular details regarding surgical technique and hemostatic strategies were not available, which could have impacted bleeding and transfusion outcomes.

## Conclusions

In patients undergoing urgent CABG after recent ACS, continuation of DAPT within 48 hours preoperatively is associated with increased rate of reinterventions, transfusion, and mortality. These findings highlight the potential harm of continuing DAPT up to the time of surgery.

Importantly, we observed substantial variation in preoperative DAPT use across Dutch heart centers, reflecting a lack of standardized perioperative protocols. This practice heterogeneity underscores the current uncertainty in clinical decision-making and the urgent need for more precise, evidence-based guidelines tailored to this high-risk population. Prospective multicenter studies are needed to confirm these findings and define optimal antiplatelet strategies, carefully balancing bleeding and ischemic risks.

## Conflict of Interest Statement

The authors reported no conflicts of interest.

The *Journal* policy requires editors and reviewers to disclose conflicts of interest and to decline handling or reviewing manuscripts for which they may have a conflict of interest. The editors and reviewers of this article have no conflicts of interest.

## References

[1] Byrne R.A., Rossello X., Coughlan J.J., Barbato E., Ibanez B., ESC Scientific Document Group (2023). 2023 ESC guidelines for the management of acute coronary syndromes. Eur Heart J.

[2] Joint Committee on Clinical Practice Guidelines (2025). 2025 ACC/AHA/ACEP/NAEMSP/SCAI guideline for the management of patients with acute coronary syndromes. J Am Coll Cardiol.

[3] Borovac J.A., Ferri-Certic J., Miric D. (2023). Revascularization with coronary artery bypass grafting in non-ST-elevation acute coronary syndromes: a snapshot of randomized trials and registries. Arq Bras Cardiol.

[4] Neumann J.T., Goßling A., Sörensen N.A., Blankenberg S., Magnussen C., Westermann D. (2020). Temporal trends in incidence and outcome of acute coronary syndrome. Clin Res Cardiol.

[5] Yusuf S., Zhao F., Mehta S.R. (2001). Effects of clopidogrel in addition to aspirin in patients with acute coronary syndromes without ST-segment elevation. N Engl J Med.

[6] Chen L., Bracey A.W., Radovancevic R. (2004). Clopidogrel and bleeding in patients undergoing elective coronary artery bypass grafting. J Thorac Cardiovasc Surg.

[7] Nagashima Z., Tsukahara K., Uchida K. (2017). Impact of preoperative dual antiplatelet therapy on bleeding complications in patients with acute coronary syndromes who undergo urgent coronary artery bypass grafting. J Cardiol.

[8] Agarwal S., Choi S.W., Fletcher S.N., Klein A.A., Gill R., Contributors (2021). The incidence and effect of resternotomy following cardiac surgery on morbidity and mortality. Anaesthesia.

[9] Ruel M., Chan V., Boodhwani M. (2017). How detrimental is reexploration for bleeding after cardiac surgery?. J Thorac Cardiovasc Surg.

[10] Sadeghi R., Haji Aghajani M., Miri R. (2021). Dual antiplatelet therapy before coronary artery bypass grafting in patients with myocardial infarction: a prospective cohort study. BMC Surg.

[11] Kremke M., Tang M., Bak M. (2013). Antiplatelet therapy at the time of coronary artery bypass grafting: a multicentre cohort study. Eur J Cardiothorac Surg.

[12] Hensley N.B., Holmes S.D., Metkus T.S. (2022). Center variation in use of preoperative dual antiplatelet therapy and platelet function testing at the time of coronary artery bypass grafting in Maryland. Anesth Analg.

[13] Janssen P.W.A., Claassens D.M.F., Willemsen L.M., Bergmeijer T.O., Klein P., Ten Berg J.M. (2017). Perioperative management of antiplatelet treatment in patients undergoing isolated coronary artery bypass grafting in Dutch cardiothoracic centres. Neth Heart J.

[14] Timmermans M.J.C., Houterman S., Daeter E.D. (2022). Using real-world data to monitor and improve quality of care in coronary artery disease: results from the Netherlands heart registration. Neth Heart J.

[15] Derks L., Medendorp N.M., Houterman S. (2024). Building a patient centred nationwide integrated cardiac care registry: intermediate results from the Netherlands. Neth Heart J.

[16] Houterman S., van Dullemen A., Versteegh M. (2023). Data quality and auditing within the Netherlands heart registration: using the PCI registry as an example. Neth Heart J.

[17] Qu J., Zhang D., Zhang H. (2022). Preoperative clopidogrel and outcomes in patients with acute coronary syndrome undergoing coronary artery bypass surgery. J Thorac Cardiovasc Surg.

[18] Schoerghuber M., Kuenzer T., Biancari F. (2024). Platelet inhibitor withdrawal and outcomes after coronary artery surgery: an individual patient data meta-analysis. Eur J Cardiothorac Surg.

[19] Alström U., Levin L.Å., Ståhle E., Svedjeholm R., Friberg O. (2012). Cost analysis of re exploration for bleeding after coronary artery bypass graft surgery. Br J Anaesth.

